# LePrimAlign: local entropy-based alignment of PPI networks to predict conserved modules

**DOI:** 10.1186/s12864-019-6271-3

**Published:** 2019-12-24

**Authors:** Sawal Maskey, Young-Rae Cho

**Affiliations:** 1 Department of Computer Science, Baylor University, One Bear Place #97141, Waco, 76798 TX USA; 2 Bioinformatics Program, Baylor University, One Bear Place #97141, Waco, 76798 TX USA

**Keywords:** Network alignment, Local network alignment, PPI networks, Protein-protein interactions, Conserved modules, Protein complex prediction

## Abstract

**Background:**

Cross-species analysis of protein-protein interaction (PPI) networks provides an effective means of detecting conserved interaction patterns. Identifying such conserved substructures between PPI networks of different species increases our understanding of the principles deriving evolution of cellular organizations and their functions in a system level. In recent years, network alignment techniques have been applied to genome-scale PPI networks to predict evolutionary conserved modules. Although a wide variety of network alignment algorithms have been introduced, developing a scalable local network alignment algorithm with high accuracy is still challenging.

**Results:**

We present a novel pairwise local network alignment algorithm, called LePrimAlign, to predict conserved modules between PPI networks of three different species. The proposed algorithm exploits the results of a pairwise global alignment algorithm with many-to-many node mapping. It also applies the concept of graph entropy to detect initial cluster pairs from two networks. Finally, the initial clusters are expanded to increase the local alignment score that is formulated by a combination of intra-network and inter-network scores. The performance comparison with state-of-the-art approaches demonstrates that the proposed algorithm outperforms in terms of accuracy of identified protein complexes and quality of alignments.

**Conclusion:**

The proposed method produces local network alignment of higher accuracy in predicting conserved modules even with large biological networks at a reduced computational cost.

## Background

The genome-wide study of proteins has considered the whole set of relationships between them on a system level as they form a complex network of interactions. A graph-theoretic model is commonly used to represent a set of protein-protein interactions (PPIs). A PPI network is a graph *G*=(*V*,*E*) where *V* is a set of labeled nodes representing proteins and *E* is a set of edges representing interactions between proteins.

Recent research in this area has focused on systematic comparison of PPI networks of different organisms. This type of computational analysis is called network alignment. The network alignment problem includes finding the entire mapping of nodes and conserved edges between the mapped node pairs within two or more networks. This problem can be applied to PPI networks because interactions between proteins are ideally conserved across species. Identifying conserved interaction patterns provides a significant insight into the principles deriving evolution of cellular organizations and their functions [1].

Network alignment is a computationally NP-hard problem owing to NP-completeness of the underlying subgraph isomorphism problem [2]. Hence, heuristic approaches for solving the network alignment problem should be sought. Various network alignment algorithms [3, 4] have been proposed to approximate solutions heuristically. The existing algorithms can be classified into pairwise and multiple network alignments according to the number of networks to be aligned. Pairwise network alignment aligns two networks, whereas multiple network alignment aligns three or more networks simultaneously.

The network alignment algorithms can also be classified into local and global network alignments based on the target region of interests. Global network alignment deals with aligning entire networks and finding the maximal set of mapped node pairs. Local network alignment, on the other hand, searches for highly similar sub-networks that likely represent conserved substructures. From a biological perspective, global network alignment seeks a comprehensive functional mapping of proteins between species while local network alignment identifies evolutionary conserved modules or protein complexes. Sometimes, local alignment is considered as many-to-many node mapping such that a node from one network can be aligned to multiple nodes from the other network, whereas global alignment as one-to-one node mapping with pairing all nodes from the smaller network. However, we have observed that some global alignment algorithms [5–7] produce many-to-many node mapping and do not connect all nodes from the smaller network.

One of the earliest global alignment algorithms IsoRank [8] estimates the node correspondence using a modification of the PageRank algorithm [9], where the basic idea is that two proteins have high probability to be aligned if their neighbors are matched well. IsoRankN [10] is an extension of IsoRank to align multiple PPI networks by using a spectral clustering method called PageRank-Nibble [11]. Both IsoRank and IsoRankN are relatively time consuming and require a large amount of memory as the network size increases. SMETANA [5] and CUFID [12] perform a Markov random walk in interconnected networks to compute steady-state distribution. CUFID applies a bipartite matching to obtain one-to-one node mapping, whereas SMETANA allows many-to-many node mapping. PrimAlign [7] models the interconnected PPI network pair as a Markov chain that is iteratively transited until convergence. This model is combined with the principles of PageRank and sparse computation. Several recent global alignment algorithms such as MANGNA [13], MAGNA++ [14], and SANA [15] use random search algorithms to optimize an objective function. MAGNA optimizes an edge conservation measure using a genetic algorithm. MAGNA++ maximizes both edge conservation and node conservation measures. SANA optimizes an edge conservation measure called the Symmetric Substructure Score (S3) using simulated annealing. ModuleAlign [16] computes an alignment score by combining the homology and topology scores, and then iteratively selects the highest-scoring protein pairs by an optimal bipartite matching. PROPER [17] employs the percolation graph matching to align input networks using the network structures and the seeds generated by sequence similarities. Fuse [18] is a multiple global network alignment algorithm that computes protein similarity scores using the non-negative matrix tri-factorization method to predict associations between proteins whose homology and functional similarity are supported by all networks.

PathBLAST [19], one of the earliest local network alignment tools, identifies conserved pathways by pairing interactions between orthologous proteins. It takes a pathway in a query, aligns it to a PPI network, and outputs all matching paths from the network. NetworkBLAST [20] is an upgraded version of PathBLAST which aligns two networks by searching for highly similar subnetworks and extends them in a greedy fashion. A recent pairwise local alignment method, AlignMCL [21] builds a weighted alignment graph by merging two networks based on orthologous protein pairs and weighting the edges by reliability of alternative paths. Similar sub-networks are identified by performing Markov Clustering in the alignment graph. LocalAli [22] is another local network alignment tool that can identify functionally conserved modules in multiple networks. It constructs evolution history of the modules based on the maximum parsimony evolutionary model and identifies the conserved modules which have been evolved from a common ancestral module through a series of evolutionary events.

In this paper, we propose a new pairwise local network alignment method called LePrimAlign - Local Entropy-based PageRank-inspired Markovian Alignment which uses graph-theoretic principles and the results of a many-to-many global network alignment algorithm to identify a set of conserved substructures between two PPI networks. To compare the performance of this approach with state-of-the-art local network alignment methods such as NetworkBLAST, AlignMCL and LocalAli, we have used the human, yeast and fruit-fly PPI networks in a genomic scale.

## Result

### LePrimAlign

**Fig. 1:**
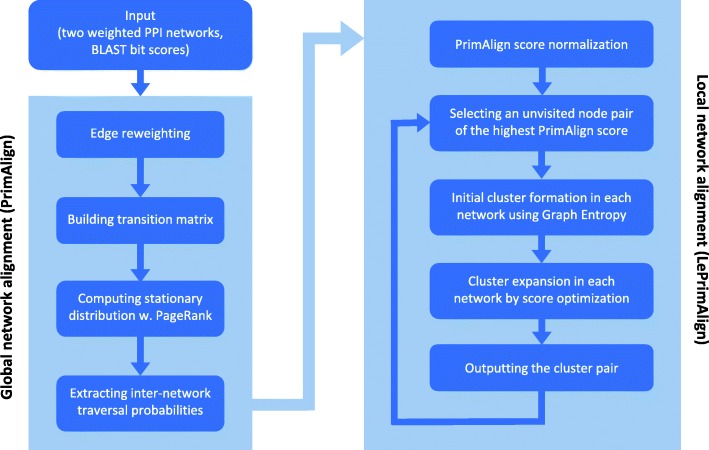
**The overall flow diagram of LePrimAlign** The proposed LePrimAlign algorithm takes two weighted PPI networks and BLAST scores of inter-network protein pairs as input, implements global network alignment PrimAlign as preprocessing, normalizes the PrimAlign scores, and iteratively performs four main steps for local network alignment: (1) seed node selection, (2) initial cluster formation, (3) cluster expansion, and (4) outputting the cluster pair

The proposed pairwise local network alignment algorithm, LePrimAlign, properly integrates a powerful global alignment algorithm with the graph-theoretic concept and the optimization process of local alignment by cluster expansion to identify conserved modules. Figure 1 exhibits the flow chart showing the entire process of LePrimAlign. The proposed algorithm performs PrimAlign as preprocessing. According to the global alignment scores of protein pairs between two networks, it selects the seed node pairs and forms the pairs of initial clusters based on the concept of Graph Entropy. Each cluster pair is expanded to optimize the local network alignment scores in a combination of intra-network and inter-network scores. The set of aligned cluster pairs is finally returned by this algorithm as output. The theoretical details of LePrimAlign will be described in the Method section.

### Data acquisition

In this study, the PPI networks of human (*Homo sapiens*), yeast (*Saccharomyces cerevisiae*) and fruit fly (*Drosophila melanogaster*) were used to evaluate the proposed approach as they are well-explored. The up-to-date genome-scale PPI data have been downloaded from BioGRID [23] and filtered for physical interactions. The interacting proteins were paired with genes that they are produced by, and maintained and treated as gene-to-gene interactions. The PPI networks obtained contain over 269,000 interactions for human with more than 15,800 unique genes, over 88,000 interactions for yeast with almost 5,800 unique genes, and over 60,000 interactions for fruit fly with more than 9,260 unique genes.

To weight the edges in each PPI network, we used semantic similarity (SS). We assumed that the higher semantic similarity two proteins are, the more confident an interaction between them is. We used simGIC [24] to measure the semantic similarity between proteins within each PPI network. The ontology and its annotation files were downloaded from the GO database [25].

The sequence similarity can be either BLAST bit-score or BLAST e-value. We have used the data set bundled with PrimAlign [7] for the BLAST bit-score and BLAST e-value. This file contains the sequence similarity scores over 55,000 human-yeast gene pairs, over 39,000 human-fruit fly gene pairs, and around 8,800 yeast-fruit fly gene pairs.

The protein complex data sets that have been determined by small-scale and large-scale experiments are helpful for evaluating accuracy of the modules aligned by local network alignment algorithms. A total of 2,576 distinct protein complexes for human were obtained from CORUM [26] and PCDq [27]. For yeast, we used 734 protein complexes from CYC2008 [28]. For fruit fly, we considered 556 protein complexes from the DPiM data set [29].

### Experimental setup

We have compared the performance of the proposed local network alignment algorithm with NetworkBLAST [20], AlignMCL [21], and LocalAli [22]. Since LocalAli is a multiple network alignment method and the others are pairwise network alignment methods, we have aligned only two PPI networks at a time for LocalAli. For AlignMCL and LePrimAlign, we have used - log of BLAST e-value for sequence similarity while BLAST bit-scores were used for NetworkBLAST and LocalAli.

**Fig. 2:**
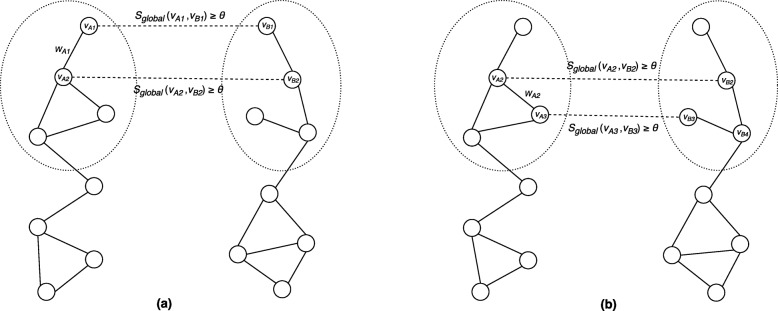
**A schematic view of (a) a match and (b) a gap between two clusters in different PPI networks** In this example, *θ* denotes the PrimAlign score threshold to select the node pairs as seeds for local network alignment. A match represents an edge in one network directly conserved in the other whereas a gap represents an edge in one network indirectly conserved in the other

The evaluation metrics described in the Method section were used to compare the performance. LePrimAlign has three user-configurable parameters: the threshold *θ* of global alignment scores for selecting seed pairs as shown in Fig. 2, the gap penalty *β* in Formula (12), and the local alignment scoring parameter *γ* in Formula (14). From our experiments, we have observed that *β* is insensitive to prediction accuracy and alignment quality. We thus set *β* to a default value of 1.0 for all the tasks. We have implemented LePrimAlign by changing the parameter values of *γ* and *θ*. The comprehensive alignment results are shown in Additional Files 1, 2 and 3. We finally set *γ* to 0.25 for all the tasks with any two PPI networks. The threshold *θ* was set to 1 for alignment between human and yeast networks and between human and fruit fly networks. For the yeast and fruit fly pair, a very small number of clusters were produced with *θ*=1 due to a smaller number of candidate seed nodes than the other pairs of PPI networks (i.e., a smaller number of known orthologs), so we used a lower threshold *θ*=0.1 for this pair of networks.

For NetworkBLAST, AlignMCL, and LocalAli, we have used default parameter values. For NetworkBLAST, the probability of an interaction within a complex was set to 0.9, and the e-value threshold for sequence similarity was 1*e*−30. AlignMCL requires only one parameter, the inflation parameter to implement Markov Clustering. We used 2.8 for this inflation parameter as default. LocalAli asks to set up many parameter values. The first and second impact factors of the evolutionary rate were set to 0.2 and 2.0, respectively. The score threshold of the sub-networks to be qualified was 0.2 with the seed size of 2. The minimal and maximal numbers of extension were set to 3 and 13, respectively.

### Comparison with known modules

Table 1 shows the numbers of clusters generated by four local network alignment algorithms for three different pairs of PPI networks. NetworkBLAST has delivered unusual results. For large, complex networks such as the human-yeast PPI network pair, NetworkBLAST produced an extremely large number of clusters whereas it produced a very small number of clusters for smaller networks such as the yeast-fruit fly network pair. This indicates NetworkBLAST is very sensitive to topological complexity and the amount of interconnections of input networks. However, LePrimAlign produced relatively consistent numbers of clusters for any pairs of PPI networks although these numbers are smaller than the numbers of clusters produced by AlignMCL and LocalAli.

**Table 1:** Comparison of local network alignment results of three previous algorithms and the proposed method and their *f*-scores as prediction accuracy of conserved protein complexes

	**Human-yeast network alignment**				**Human-fruit fly network alignment**				**Yeast-fruit fly network alignment**			
	**Num. of clusters**		***f****-score*		**Num. of clusters**		***f****-score*		**Num. of clusters**		***f****-score*	
**Algorithms**	**Human**	**Yeast**	**Human**	**Yeast**	**Human**	**Fruit fly**	**Human**	**Fruit fly**	**Yeast**	**Fruit fly**	**Yeast**	**Fruit fly**
NetworkBlast	1626	1695	0.431	0.428	1162	925	0.348	0.174	70	65	0.411	0.315
AlignMCL	926	838	0.285	0.285	683	681	0.279	0.161	309	309	0.279	0.176
LocalAli	400	400	0.164	0.172	400	400	0.170	0.121	384	384	0.171	0.133
LePrimAlign	180	175	0.418	0.441	201	179	0.430	0.253	110	105	0.352	0.235

Three different pairs of PPI networks have been aligned: the human and yeast PPI network pair, the human and fruit fly PPI network pair, and the yeast and fruit fly PPI network pair

Table 1 also shows the average *f*-scores of the clusters compared to known protein complexes. LePrimAlign and NetworkBLAST achieved higher average *f*-scores for all the pairs of PPI networks than AlignMCL and LocalAli. AlignMCL could generate a relatively large number of clusters but had lower accuracy of predicted protein complexes than NetworkBLAST and LePrimAlign. LocalAli had more stable results with nearly the same number of clusters and similar accuracy across all network pairs. However, LocalAli had the lowest *f*-scores among its competitors. LePrimAlign had higher accuracy than NetworkBLAST when aligning the human and fruit fly PPI networks whereas NetworkBLAST was better than LePrimAlign for the yeast and fruit fly networks. In LePrimAlign, the threshold *θ* value could be leveraged for increasing the number of clusters or improving their accuracy, as shown in Additional Files 1, 2 and 3. A high value of *θ* results in a small number of clusters but high accuracy, whereas a low value of *θ* causes a large number of clusters but low accuracy.

### Alignment quality

Table 2 shows the alignment quality comparison of four local alignment algorithms for three different pairs of PPI networks in terms of the average inter-species semantic similarity (ISS), the average number of conserved edges (CE), and the average number of functionally consistent conserved edges (F-CE). These evaluation metrics are described in detail in the Method section. Overall, LePrimAlign has the highest ISS. However, NetworkBLAST has more conserved edges and more functionally consistent conserved edges than LePrimAlign when aligning human-yeast PPI networks although LePrimAlign has more when aligning the other pairs of PPI networks.

**Table 2:** Comparison of local network alignment quality of three previous algorithms and the proposed method in terms of the average inter-species semantic similarity (ISS), the average number of conserved edges (CE), and the average number of functionally consistent conserved edges (F-CE)

	**Human-yeast network alignment**			**Human-fruit fly network alignment**			**Yeast-fruit fly network alignment**		
**Algorithms**	**ISS**	**CE**	**F-CE**	**ISS**	**CE**	**F-CE**	**ISS**	**CE**	**F-CE**
NetworkBlast	0.364	30.461	26.659	0.177	16.301	10.470	0.242	5.845	5.634
AlignMCL	0.292	2.984	2.459	0.144	0.713	0.492	0.185	0.280	0.241
LocalAli	0.117	2.375	1.845	0.066	0.980	0.625	0.104	0.740	0.526
LePrimAlign	0.373	17.944	15.872	0.198	22.787	17.470	0.235	13.884	13.661

In our careful observation, most of the resultant clusters aligned by NetworkBLAST are highly overlapping. In other words, they share a large number of proteins. And, a significant amount of conserved edges occur in most of these overlaps of clusters. For fair comparison, we have removed highly overlapping clusters and compared again the number of conserved edges. To identify the degree of cluster overlaps, we have used the Jaccard index as the ratio of common proteins out of all distinct proteins in the clusters. Any clusters having the Jaccard index higher than 0.4 are considered highly overlapping. Among such clusters, only the one with the largest size is considered in the final evaluation. On removing these highly overlapping clusters, the number of clusters and the number of conserved edges that were produced by NetworkBLAST reduced significantly. However, the effects on the other algorithms were not significant. As a result, LePrimAlign has larger numbers of conserved edges and functionally consistent conserved edges than NetworkBLAST for all the experiments as shown in Table 3.

**Table 3:** Comparison of local network alignment results and alignment quality, the average numbers of conserved edges (CE) and functionally consistent conserved edges (F-CE), after removing highly overlapping clusters

	**Human-yeast network alignment**				**Human-fruit fly network alignment**				**Yeast-fruit fly network alignment**			
**Algorithms**	**Num. of clusters**		**CE**	**F-CE**	**Num. of clusters**		**CE**	**F-CE**	**Num. of clusters**		**CE**	**F-CE**
	**Human**	**Yeast**			**Human**	**Fruit fly**			**Yeast**	**Fruit fly**		
NetworkBlast	272	289	15.493	13.768	164	158	12.408	8.663	35	34	4.278	4.056
AlignMCL	826	823	3.025	2.493	681	679	3.025	2.493	307	307	0.280	0.244
LocalAli	400	400	2.375	1.845	400	400	0.980	0.625	384	384	0.740	0.526
LePrimAlign	161	160	17.469	15.216	201	179	22.787	17.470	110	105	13.884	13.661

### Runtime evaluation

To evaluate the runtime performance, we executed each individual algorithm with default parameters on an Intel(R) Core(TM) i5-7200U CPU with 2.50 GHz and 8 GB RAM. As shown in Table 4, AlignMCL and LocalAli were faster than the other algorithms. However, their performance in terms of accuracy and alignment quality was lower. The runtime of NetworkBLAST was the worst among the four algorithms. When aligning larger networks such as human and yeast PPI networks, NetworkBLAST ran for almost a day to return the final result. LePrimAlign, on the other hand, generated the result in approximately one hour with even better accuracy.

**Table 4:** Runtime comparison in seconds

	**Human-yeast networks**	**Human-fly networks**	**Yeast-fly networks**
NetworkBLAST	93321	12728	567
AlignMCL	975	325	34
LocalAli	193	206	112
LePrimAlign	4101	2522	830

For the proposed method LePrimAlign, the PrimAlign score threshold *θ* of 1 was used for aligning human-yeast PPI networks and human-fly PPI networks and 0.1 was used for aligning yeast-fly PPI networks

The runtime of LePrimAlign depends on the global network alignment score threshold *θ*, i.e., the number of aligned cluster pairs. For the results in Table 4, we used *θ* of 1 for aligning human-yeast PPI networks and human-fly PPI networks and 0.1 for aligning yeast-fly PPI networks. However, as a lower value of *θ* is used, its runtime decreases rapidly.

### Validation of seed selection

The proposed approach is a mixture of network alignment and graph clustering techniques. The graph clustering that was adopted in this approach follows the process of selecting seeds and expanding them to detect locally optimized clusters which match biologically significant functional modules. Such graph clustering process can have the best performance when the selected seed nodes are functionally core proteins in a PPI network.

We evaluated functional essentiality of the seed nodes mapped between networks, which were selected by the proposed algorithm. We used the sets of functionally essential genes of yeast from the DEG [30] and MIPS [31] databases. These genes have been confirmed by gene disruption experiments. We measured (1) the ratio of essential genes that were selected as seed nodes in LePrimAlign (called a true positive rate). This result was compared to (2) the ratio of essential genes to a set of interconnected genes between networks in the original input data (considered putative orthologs). For fair comparison, we randomly selected the same number of interconnected genes as the seed nodes in LePrimAlign. The essentiality of the seed nodes was also compared to (3) the ratio of essential genes to a set of nodes randomly selected in the entire PPI network. Same to above, we selected the same number of nodes as the seed nodes in LePrimAlign.

Table 5 shows the three evaluation results described above with the two different sets of essential genes of yeast. The ratios of essential genes to the randomly selected nodes in the entire yeast PPI network were 30% and 28%. When we considered only the yeast genes interconnected with genes in the other species by high sequence similarity (i.e., the genes interconnected before preprocessing of LePrimAlign), the ratios of essential genes increased to 44% and 42%. When we used only the yeast genes that were mapped by global alignment scores greater than *θ* after PrimAlign implementation (i.e., the seed nodes in LePrimAlign after preprocessing), the ratios of essential genes even more increased to 59% and 57%. These results justify that the preprocessing step using the global alignment would enhance functional module prediction accuracy of the proposed local alignment.

**Table 5:** The ratios of essential genes to the seed nodes selected in the proposed local network alignment (after preprocessing), the ratio of essential genes to the genes interconnected between networks (before preprocessing), and the ratio of essential genes to randomly selected nodes in a PPI network

**essential gene**	**seed nodes selected**	**interconnected nodes**	**random nodes in a network**
**data set**	**after preprocessing**	**before preprocessing**	
DEG data set	0.586	0.445	0.305
MIPS data set	0.566	0.420	0.278

The sets of essential genes were obtained from DEG and MIPS

## Discussion

Our experiments have demonstrated that the proposed LePrimAlign algorithm predicts conserved protein complexes more accurately and generates higher-quality alignment for any PPI network pairs than three prevalent local network alignment algorithms. Although NetworkBLAST produces accurate clusters, it has two major drawbacks. First, NetworkBLAST is not scalable with very large networks. When aligning genome-wide PPI networks of human and yeast, it ran over 24 hours to receive a complete result. Apart from this computational issue, NetworkBLAST produces a large number of highly overlapping clusters. Hence, additional filtering is required on the output set. AlignMCL and LocalAli are very efficient in terms of runtime; however, their prediction of protein complexes is inaccurate. Their average inter-species semantic similarity between aligned clusters is also comparatively low.

LePrimAlign, on the other hand, generates higher-quality local network alignment at a reduced computational cost. This algorithm requires several parameters that a user needs to specify: the threshold of global alignment scores *θ*, the gap penalty *β*, and the scoring parameter *γ*. Out of these parameters, the number of aligned clusters, prediction accuracy, and runtime mostly depend on *θ*. Similar to PrimAlign, a higher value of *θ* (e.g., *θ*>1.5) results in higher accuracy but fewer clusters. A higher threshold is likely to miss some correct protein complex alignments (i.e., more false negatives). On the other hand, a lower value of *θ* (e.g., *θ*<0.5) is likely to produce a large number of clusters with relatively lower accuracy, selecting some incorrect protein complex alignments (i.e., more false positives). The large number of clusters generated also means longer running time. The *θ* value of 0.5 on large networks such as the human-yeast PPI network pair can take up to 100 minutes for achieving a complete local alignment result.

Although LePrimAlign outperforms the previous local network alignment algorithms, all the methods have relatively low accuracy in protein complex prediction. As shown in Table 1, all the *f*-scores achieved in our experiment are less than 0.5. The low *f*-scores were caused by very low precision. The cogent reason for such low precision would be that the ground-truth data sets include a large amount of false negatives, i.e., actual protein complexes that do not exist in the ground-truth data sets. As ground-truth, we used 734 yeast protein complexes, 2,576 human protein complexes, and 556 fruit fly protein complexes, which were obtained from the largest databases or were combined from multiple databases. However, we still do not expect that these ground-truth data sets have complete coverage of actual protein complexes.

Although the local network alignment algorithms show low *f*-scores in protein complex prediction in our experiment, they still have higher accuracy than graph clustering algorithms to predict protein complexes from a single PPI network. For this comparison, we tested two graph clustering algorithms, the Graph-Entropy algorithm [32] which is based on a similar technique to LePrimAlign and the Markov Clustering algorithm (MCL) [33] which is the most popular and applied to the previous network alignment algorithm AlignMCL. We used the same PPI networks of human, yeast and fruit fly, and the same ground-truth data sets of protein complexes that were used for our evaluation of local network alignment algorithms. Table 6 shows the *f*-scores of these graph clustering algorithms on each PPI network. As compared to the *f*-scores in Table 1, it is apparent that LePrimAlign has higher accuracy than the selected single-graph clustering methods.

**Table 6:** The single-graph clustering results and their *f*-scores in protein complex prediction by two graph clustering algorithms

	**human network**		**yeast network**		**fruit fly network**	
**algorithms**	**num. of clusters**	***f****-scores*	**num. of clusters**	***f****-scores*	**num. of clusters**	***f****-scores*
Graph-Entropy	1061	0.211	223	0.170	1592	0.139
MCL	2351	0.220	580	0.372	1156	0.184

These algorithms had lower *f*-scores than the proposed local network alignment algorithm LePrimAlign when compared to the *f*-scores in Table 1

## Conclusion

Local network alignment algorithms for biological networks aim to identify pairs of conserved modules. Identifying such modules helps understanding the principles deriving evolution in a system level. Since network alignment identifies a comprehensive functional mapping of proteins between species, it also provides an efficient way of predicting functions of unknown proteins and completing functional annotations especially in less-studied species. However, because it is a computationally NP-hard problem, this task should be performed using heuristics to make scalable for very large, genome-wide biological networks.

In this paper, we have presented a novel pairwise local network alignment algorithm based on the ideas of the global network alignment PrimAlign, the entropy-based graph clustering, and optimizing the local alignment score in a combination of intra-network and inter-network scores. The proposed method outperformed the existing algorithms in terms of the accuracy of predicted complexes and the alignment quality. Compared to some recent local network alignment algorithms such as AlignMCL and LocalAli, only one limitation of LePrimAlign might be the runtime on extremely large networks. It takes up 100 minutes for the genome-wide PPI networks of human and yeast with the threshold *θ* of 0.5. The current implementation of LePrimAlign runs on a single thread only. We can improve the runtime performance by processing the seed node pairs on multiple threads in a parallel manner because the step of initial cluster formation and cluster expansion for each pair of seed nodes is independent of each other.

## Method

### Graph entropy

Graph Entropy is a metric based on information theory to assess modularity of a graph [32]. Let us consider an undirected graph *G*(*V*,*E*) that is decomposed into a set of clusters. A cluster is considered an induced subgraph *C*(*V*_*C*_,*E*_*C*_) on *G* that has dense intra-connections and sparse interconnections. Given a cluster *C*(*V*_*C*_,*E*_*C*_), an inner link of a node *v* is defined as the edge from *v* to the node in *V*_*C*_ and an outer link of *v* is defined as the edge from *v* to the node not in *V*_*C*_. If *G* is an unweighted graph, then we can define the probability of *v* having inner links as:

1 $$ p_{i}(v) = \frac{|V_{C}\cap N(v)|}{|N(v)|}  $$

where *N*(*v*) is the set of neighboring nodes of *v* and |*N*(*v*)| is the total number of nodes in *N*(*v*). If *G* is a weighted graph, we can define the probability of *v* having inner links as:

2 $$ p_{i}(v) = \frac{\sum\limits_{v_{c} \in V_{C}} w(v,v_{c})}{\sum\limits_{v' \in N(v)} w(v,v')}  $$

where *w*(*v*,*v*^′^) is the weight of the edge between *v* and *v*^′^. The probability of *v* having outer links is then computed by

3 $$ p_{o}(v) = 1 - p_{i}(v)  $$

Given a cluster *C*(*V*_*C*_,*E*_*C*_), we can define the node entropy *e*(*v*) based on the probability distribution of its inner links and outer links as:

4 $$ e(v) = -p_{i}(v)\log_{2}p_{i}(v) - p_{o}(v)\log_{2}p_{o}(v)  $$

The entropy of graph *G*(*V*,*E*) is then computed by the sum of the entropy of all the nodes in *G*.

5 $$ e(G) = \sum\limits_{v \in V} e(v)  $$

A graph with lower graph entropy indicates that the nodes inside the current cluster have more inner links and less outer links, and the nodes outside the cluster have more outer links and less inner links.

### PrimAlign

PrimAlign - PageRank-Inspired Markovian Alignment [7] is a pairwise global network alignment algorithm for many-to-many node mapping. It was built upon the idea of modeling two interconnected networks as a Markov chain and combining this model with the basic principles of the original PageRank algorithm and sparse computation.

As input, PrimAlign takes two weighted PPI networks *G*_1_ and *G*_2_ to be aligned and a list of sequence similarity scores of protein pairs between the networks. Edge weights in each PPI network represent the confidence of the interactions. A transition matrix *T* is constructed where each element of the matrix is either an edge weight within each network or a weighted sequence similarity score between the networks. The matrix is normalized such that each row sums to 1.

6 $$ T = \left[ \begin{array}{cc} T_{G_{1}\rightarrow G_{1}} & T_{G_{1}\rightarrow G_{2}} \\ T_{G_{2}\rightarrow G_{1}} & T_{G_{2}\rightarrow G_{2}} \end{array} \right]  $$

where $T_{G_{1}\rightarrow G_{1}}$ and $T_{G_{2}\rightarrow G_{2}}$ are the partial matrices for transitions within each network built from edge weights. $T_{G_{1}\rightarrow G_{2}}$ and $T_{G_{2}\rightarrow G_{1}}$ are the partial matrices for transitions between the networks built from weighted sequence similarity scores.

Once the transition matrix is constructed, the PageRank algorithm is performed iteratively to calculate the stationary distribution of nodes. In each iteration, the probability distribution is updated using Formulas (7) and (8) until convergence.

7 $$ p^{temp} = \alpha p^{(t)}T - (\alpha p^{(t)}q + 1 - \alpha) \frac{u}{n}  $$

8 $$ p^{(t+1)} = \frac{p^{temp}}{\| p^{temp} \|_{1}}  $$

where *α* is a damping factor; *q* is the column vector for each row of *T*: 1 if the row has all 0s and 0 otherwise; *u* is the row vector of 1s; *p*^(*t*)^ is the state probability distribution vector at step *t*.

For each node pair, *v*_1_ and *v*_2_, between two networks, *G*_1_ and *G*_2_, the global alignment score *S*_*global*_(*v*_1_,*v*_2_) is calculated using the traversal probabilities as shown below.

9 $$ \begin{aligned} S_{global}(v_{1},v_{2}) &= \left(p(v_{1}) \frac{T_{G_{1}\rightarrow G_{2}}[v_{1},v_{2}]}{\|T_{G_{1}\rightarrow G_{2}}[v_{1},1:n_{G_{2}}]\|_{1}}\right.\\ &\left.\quad+ p(v_{2})\frac{T_{G_{2}\rightarrow G_{1}}[v_{1},v_{2}]}{\|T_{G_{2}\rightarrow G_{1}}[v_{2},1:n_{G_{1}}]\|_{1}}\right)n \end{aligned}  $$

where $n_{G_{1}}$ and $n_{G_{2}}$ are the total number of nodes in *G*_1_ and *G*_2_ respectively, and $n = n_{G_{1}} + n_{G_{2}}$.

### LePrimAlign

LePrimAlign - Local Entropy-Based PrimAlign is the newly proposed pairwise local network alignment algorithm. As shown in Fig. 1, this algorithm takes the results of PrimAlign and applies local search for optimal conserved modules based on a new scoring scheme.

#### Input

The expected inputs of this algorithm are similar to PrimAlign [7]. It requires three input files. The first two files are the weighted PPI networks of two species that are to be aligned. Edge weights in each PPI network represent the confidence of the interactions. For an edge weight in our experiments, we computed the semantic similarity score of the interacting proteins by simGIC. The third file contains sequence similarity scores such as - log of BLAST e-value between proteins from different networks.

#### Preprocessing

The global network alignment algorithm PrimAlign is performed as preprocessing. The new scores of inter-network node pairs are calculated by stationary-distributed transition probabilities. We finally extract the transition probabilities of all connected node pairs between two PPI networks.

#### Score normalization

The initial step of the main process is to normalize the PrimAlign scores such that they strictly lie between 0 and 1, inclusive. Since a few scores are very high (i.e., greater than 10) but majority are low (i.e., less than 1), we used log normalization as follows:

10 $$ S_{\text{norm}}(v_{1},v_{2}) = \log_{b} (1 + S_{global}(v_{1},v_{2}))  $$

where

11 $$ b = \big\lceil 1 + \max\limits_{v_{i} \in G_{1}, v_{j} \in G_{2}} S_{global}(v_{i},v_{j}) \big\rceil  $$

#### Initial clusters formation

The next step is to form an initial cluster on each network. After preprocessing, each pair of mapped proteins is selected as seed nodes iteratively in descending order of their global network alignment scores until the score of a pair falls below a threshold *θ*. If both seed nodes in two PPI networks are visited, then we pick the next pair of seed nodes.

We have used the same process as entropy-based graph clustering [32] for initial cluster formation. For each of the seed nodes, an initial cluster is formed by adding the seed node and all its neighbors. Then a neighbor with the highest weighted degree is removed from the initial cluster only if graph entropy decreases. We perform this task iteratively in decreasing order of weighted degree until all neighbors are processed. We finally obtain an initial cluster having the lowest graph entropy in each network.

#### Clusters expansion

In this step, we expand the initial clusters to generate putative conserved modules. At first, we calculate the alignment score between the two initial clusters. The alignment score is a linear combination of two different scoring metrics, namely (a) an intra-network score and (b) an inter-network score. For the intra-network score, we define a match and a gap for an edge pair between two clusters as shown in Fig. 2. A match is the case when an edge in the first cluster is directly conserved in the second cluster as shown in Fig. 2(a). A gap is the case when an edge in the first cluster is indirectly conserved with an unaligned node (i.e., the node *v*_*B*4_ in Fig. 2(b)) between two aligned nodes in the second cluster. After identifying the edges with match or gap between two clusters *C*_1_ and *C*_2_, we calculate the intra-network score of *C*_1_ as:

12 $$ \begin{aligned} S_{intra}(C_{1}, C_{2}) &= \frac{1}{|E_{1}|}\left(\sum\limits_{(v_{i}, v_{j}) \in match \subset E_{1}} w(v_{i}, v_{j})\right.\\ &\left.\quad + \beta \sum\limits_{(v_{i}, v_{j}) \in gap \subset E_{1}} w(v_{i}, v_{j}) \right) \end{aligned}  $$

where *E*_1_ is the set of edges in *C*_1_, |*E*_1_| is the size of *E*_1_, *w*(*v*_*i*_,*v*_*j*_) is the weight of the edge (*v*_*i*_,*v*_*j*_)∈*E*_1_, and *β* is a parameter to penalize gaps where 0≤*β*≤1.

The inter-network score is calculated by averaging the best normalized global alignment scores of the nodes in the first cluster that are aligned to any nodes in the second cluster. The normalized global alignment scores used here include the scores of all interconnected node pairs without any threshold. This inter-network score of *C*_1_ can be formulated as:

13 $$ S_{inter}(C_{1}, C_{2}) = \frac{1}{|V_{1}|} \sum\limits_{v_{i} \in C_{1}} \max\limits_{v_{j} \in C_{2}} S_{norm}(v_{i}, v_{j})  $$

where *V*_1_ is the set of nodes in *C*_1_ and |*V*_1_| is the size of *V*_1_. The final local network alignment score of *C*_1_ is then a linear combination of the intra-network and inter-network scores.

14 $${} S_{local}(C_{1}, C_{2}) = \gamma S_{inter}(C_{1}, C_{2}) + (1-\gamma) S_{intra}(C_{1}, C_{2})  $$

where *γ* is a scoring parameter in the range of 0≤*γ*≤1. It controls the contribution of intra-network and inter-network scores.

We calculate two initial local network alignment scores, *S*_*local*_(*C*_1_,*C*_2_) and *S*_*local*_(*C*_2_,*C*_1_), between the aligned clusters *C*_1_ and *C*_2_. Then, we iteratively add the nodes on the outer boundary to each cluster if this node addition increases the score. The outer boundary nodes represent the nodes outside the cluster which have at least one link to any node inside the cluster. The iterative node addition can be done in descending order of node degree until both clusters cannot expand further. The nodes are added simultaneously to each of the aligned clusters to score *S*_*local*_(*C*_1_,*C*_2_) and *S*_*local*_(*C*_2_,*C*_1_). We have considered that an aligned cluster should have at least two proteins. If the sizes of both clusters aligned are greater than one, the aligned cluster pair are added to the output set, and all nodes in these clusters are marked as visited. Then, we select another pair of seed nodes that are not visited and repeat the steps of initial cluster formation and cluster expansion until all seed node pairs within the threshold *θ* are selected. If at least one of the aligned clusters has a final score less than 0.02, we have discarded the pair in order to prevent formation of large and uneven sized clusters.

#### Output

Two output files are generated by the proposed algorithm, one for each PPI network. Each row in these files represents a pair of putative conserved clusters. Both files contain the same number of rows; two aligned clusters are in the same row of the two output files.

### Evaluation metrics

#### Comparison with known modules

We have evaluated how well the solutions provided by the local network alignment algorithms match known protein complexes that have been confirmed by various experiments. We have used *f*-scores for this evaluation. Suppose we compare an output cluster *C* generated by a local network alignment algorithm to a known protein complex *P*_*i*_. Recall *ρ* (also called a true positive rate or sensitivity) is the ratio of common proteins between *C* and *P*_*i*_ to the number of proteins in *P*_*i*_.

15 $$ \rho = \frac{|C \cap P_{i}| }{|P_{i}|}  $$

Precision *π* (also called a positive predictive value) is the ratio of common proteins between *C* and *P*_*i*_ to the number of proteins in *C*.

16 $$ \pi = \frac{|C \cap P_{i}|}{|C|}  $$

The *f*-score is then the harmonic mean of recall and precision.

17 $$ f\text{-score} = \frac{2\pi\rho}{\pi + \rho}  $$

The *f*-score ranges in the interval [0,1], with 1 corresponding to perfect prediction. This measure makes a direct comparison between an output cluster and a known protein complex without any bias towards cluster size. For each output cluster, the best match to a protein complex was obtained in regard to its *f*-score. The average *f*-score of the best matches across all output clusters was used to determine accuracy of the local network alignment result.

#### Inter-species semantic similarity

Semantic similarity measures can quantify the functional similarity between genes or gene products by comparing the ontology terms that annotate them [34]. Over the last decade, a wide range of semantic similarity measures have been introduced [35–37]. Most of these methods have been tested using GO and its annotation data sets [25].

A pair of aligned clusters are expected to have similar functions as conserved modules and hence they are likely to have high semantic similarity. As a measure of alignment quality, we have used inter-species semantic similarity. Let us consider a pair of aligned clusters *C*_1_ and *C*_2_ of two different species. Then, the inter-species semantic similarity (ISS) between *C*_1_ and *C*_2_ is defined as:

18 $$ ISS(C_{1}, C_{2}) = \frac{\sum\limits_{v_{i} \in C_{1}} \sum\limits_{v_{j} \in C_{2}} SS(v_{i}, v_{j})}{|C_{1}||C_{2}|}  $$

where *SS*(*v*_*i*_,*v*_*j*_) is the semantic similarity between proteins *v*_*i*_ and *v*_*j*_, and |*C*_1_| and |*C*_2_| are the numbers of proteins in *C*_1_ and *C*_2_, respectively. The inter-species semantic similarity ranges in the interval [0,1], with 1 corresponding to the highest functional similarity.

Among a variety of semantic similarity measures, we have used simGIC [24] as it has been demonstrated to be one of the most efficient and accurate methods to estimate functional similarity between two proteins. The simGIC scores also range between 0 and 1. The overall alignment quality of a local network alignment algorithm was determined by the average of the inter-species semantic similarity of all aligned cluster pairs.

#### Numbers of conserved edges and functionally consistent conserved edges

A conserved edge (CE) is defined as an edge *e* in one network that is directly aligned to an edge *e*^′^ in the other network where the two proteins linked by *e* have high sequence similarity (i.e., orthologs) with the two proteins linked by *e*^′^, respectively. The larger number of conserved edges between aligned clusters indicates higher accuracy in predicting conserved modules. If an edge in one network is conserved with more than one edge in the other network, then they are counted as distinct conserved edges. The average number of conserved edges across all aligned cluster pairs was used to evaluate the alignment quality of local network alignment algorithms.

We have also measured the average number of functionally consistent conserved edges for further evaluation of alignment quality. A functionally consistent conserved edge (F-CE) is defined as a conserved edge *e* in one network that is aligned to an edge *e*^′^ in the other network where the two proteins linked by *e* have high sequence similarity and high semantic similarity (SS) with the two proteins linked by *e*^′^, respectively. As the high semantic similarity condition, we have considered the simGIC scores greater than 0.2.

## Supplementary information

**Additional file 1** Comprehensive evaluation results of LePrimAlign for human and yeast PPI network alignment. The proposed LePrimAlign algorithm has been implemented by changing the parameter values: the threshold *θ* and the scoring parameter *γ*. Complex prediction accuracy and alignment quality including inter-species semantic similarities (ISS), the average number of conserved edges (CE) and the average number of functionally consistent conserved edges (F-CE) are shown.

**Additional file 2** Comprehensive evaluation results of LePrimAlign for human and fruit Fly PPI network alignment. The proposed LePrimAlign algorithm has been implemented by changing the parameter values: the threshold *θ* and the scoring parameter *γ*. Complex prediction accuracy and alignment quality including inter-species semantic similarities (ISS), the average number of conserved edges (CE) and the average number of functionally consistent conserved edges (F-CE) are shown.

**Additional file 3** Comprehensive evaluation results of LePrimAlign for yeast and fruit Fly PPI network alignment. The proposed LePrimAlign algorithm has been implemented by changing the parameter values: the threshold *θ* and the scoring parameter *γ*. Complex prediction accuracy and alignment quality including inter-species semantic similarities (ISS), the average number of conserved edges (CE) and the average number of functionally consistent conserved edges (F-CE) are shown.

## Data Availability

The source code is available at http://web.ecs.baylor.edu/faculty/cho/LePrimAlign.

## Abbreviations

CE: Conserved edges
F-CE: Functionally consistent conserved edges
ISS: Inter-species semantic similarity
PPI: Protein-protein interaction
SS: Semantic similarity
