# Supplementary material for: LePrimAlign: local entropy-based alignment of PPI networks to predict conserved modules
Source: BMC Genomics. 2019 Dec 24;20(Suppl 9):964. doi: 10.1186/s12864-019-6271-3 (PMC6929407; doi:10.1186/s12864-019-6271-3)
Supplement: Supplementary file 1 — Additional file 1 Comprehensive evaluation results of LePrimAlign for human and yeast PPI network alignment. The proposed LePrimAlign algorithm has been implemented by changing the parameter values: the threshold θ and the scoring parameter γ. Complex prediction accuracy and alignment quality including inter-species semantic similarities (ISS), the average number of conserved edges (CE) and the average number of functionally consistent conserved edges (F-CE) are shown. [file 12864_2019_6271_MOESM1_ESM.pdf]

Additional File 1. Comprehensive evaluation results of LePrimAlign for human and yeast PPI network alignment.

Threshold θ = 2	Number of clusters		Average Size		F-score (per output cluster)		Running time (sec)	Inter-species SS	Average number of CE	Average number of F-CE
	Human	Yeast	Human	Yeast	Human	Yeast				
γ = 0	93	83	3.4086	6.012	0.453202	0.474497	2998	0.430612	6.418367	4.77551
γ = 0.25	89	87	4.1798	5.6437	0.438119	0.423669	2752	0.389843	10.764045	9
γ = 0.5	84	84	3.9405	5.4167	0.446257	0.412322	2736	0.378566	9.119048	7.3333
γ = 0.75	67	67	3.9701	4.0746	0.443945	0.376276	2741	0.381668	9.268657	7.1791

Threshold θ = 1.5	Number of clusters		Average Size		F-score (per output cluster)		Running time (sec)	Inter-species SS	Average number of CE	Average number of F-CE
	Human	Yeast	Human	Yeast	Human	Yeast				
γ = 0	124	111	3.2823	5.2793	0.435237	0.464561	3592	0.447702	6.381679	4.99236
γ = 0.25	113	110	4.2655	5.6909	0.428269	0.426934	3290	0.386998	12.929204	11.2654
γ = 0.5	103	103	4.233	5.3689	0.43105	0.425047	3259	0.361402	14.737864	11.7669
γ = 0.75	90	90	4.1111	4.1444	0.426885	0.410982	3453	0.378211	16.01111	11.1444

Threshold θ = 1	Number of clusters		Average Size		F-score (per output cluster)		Running time (sec)	Inter-species SS	Average number of CE	Average number of F-CE
	Human	Yeast	Human	Yeast	Human	Yeast				
γ = 0	212	186	3.434	4.5376	0.423353	0.453596	4454	0.425926	8.477273	6.37272
γ = 0.25	180	175	4.3278	4.9486	0.417799	0.441302	4101	0.373255	17.94444	15.8722
γ = 0.5	169	166	4.568	4.8012	0.415855	0.431506	4050	0.355311	19.502959	16.7337
γ = 0.75	151	148	4.7285	4.1014	0.415598	0.418767	4315	0.354659	19.76159	14.8344

Threshold θ = 0.75	Number of clusters		Average Size		F-score (per output cluster)		Running time (sec)	Inter-species SS	Average number of CE	Average number of F-CE
	Human	Yeast	Human	Yeast	Human	Yeast				
γ = 0	310	277	3.6935	4.2635	0.409775	0.42582	5371	0.394036	10.702786	6.80805
γ = 0.25	243	236	4.8724	5.1271	0.405965	0.421311	6046	0.346535	26.2827	20
γ = 0.5	238	227	5.1597	5.1938	0.397794	0.417552	5816	0.332418	27.42437	22.668
γ = 0.75	211	204	5.237	4.5245	0.397731	0.409629	5843	0.337825	27.0283	21.7924

Threshold θ = 0.5	Number of clusters		Average Size		F-score (per output cluster)		Running time (sec)	Inter-species SS	Average number of CE	Average number of F-CE
	Human	Yeast	Human	Yeast	Human	Yeast				
γ = 0	447	398	3.7315	3.9497	0.393936	0.405124	6457	0.378463	12.286022	8.4344
γ = 0.25	360	350	5.3861	5.3943	0.374321	0.40244	5854	0.312008	38.135359	31.2624
γ = 0.5	328	317	6.0793	5.6278	0.372305	0.397916	5993	0.304256	51.50152	39
γ = 0.75	306	300	6.268	5.45	0.363969	0.386277	6089	0.298666	61.311644	43.2123
