# Supplementary material for: LePrimAlign: local entropy-based alignment of PPI networks to predict conserved modules
Source: BMC Genomics. 2019 Dec 24;20(Suppl 9):964. doi: 10.1186/s12864-019-6271-3 (PMC6929407; doi:10.1186/s12864-019-6271-3)
Supplement: Supplementary file 2 — Additional file 2 Comprehensive evaluation results of LePrimAlign for human and fruit Fly PPI network alignment. The proposed LePrimAlign algorithm has been implemented by changing the parameter values: the threshold θ and the scoring parameter γ. Complex prediction accuracy and alignment quality including inter-species semantic similarities (ISS), the average number of conserved edges (CE) and the average number of functionally consistent conserved edges (F-CE) are shown. [file 12864_2019_6271_MOESM2_ESM.pdf]

Additional File 2. Comprehensive evaluation results of LePrimAlign for human and fruit fly PPI network alignment.

**Threshold θ = 2**	**Number of clusters**		**Average Size**		**F-score (per output cluster)**		**Running time (sec)**	**Inter-species SS**	**Average number of CE**	**Average number of F-CE**
	**Human**	**Fruit Fly**	**Human**	**Fruit Fly**	**Human**	**Fruit Fly**				
γ = 0	110	90	3.6818	11.0556	0.484209	0.329056	1938	0.22557	3.801802	2.38738
γ = 0.25	81	75	3.8642	8.2667	0.492639	0.296249	1829	0.22024	7.395062	3.72839
γ = 0.5	78	75	3.8462	6.52	0.494033	0.278539	1893	0.219674	7.128205	3.85897
γ = 0.75	71	65	3.9718	5.3077	0.488174	0.259728	3194	0.200332	7.394366	4.07042

**Threshold θ = 1.5**	**Number of clusters**		**Average Size**		**F-score (per output cluster)**		**Running time (sec)**	**Inter-species SS**	**Average number of CE**	**Average number of F-CE**
	**Human**	**Fruit Fly**	**Human**	**Fruit Fly**	**Human**	**Fruit Fly**				
γ = 0	144	124	3.7083	9.3468	0.477385	0.298442	3984	0.20924	4.513699	3.11643
γ = 0.25	121	112	4.0413	7.3036	0.473496	0.288018	2272	0.21684	8.688525	5.63934
γ = 0.5	103	97	4.1748	5.8969	0.4714447	0.267679	2105	0.211307	8.653846	8.653846
γ = 0.75	95	91	4.3053	5.1868	0.470404	0.255526	2047	0.202611	9.416667	5.208333

**Threshold θ = 1**	**Number of clusters**		**Average Size**		**F-score (per output cluster)**		**Running time (sec)**	**Inter-species SS**	**Average number of CE**	**Average number of F-CE**
	**Human**	**Fruit Fly**	**Human**	**Fruit Fly**	**Human**	**Fruit Fly**				
γ = 0	229	195	4.048	7.3949	0.462557	0.253128	2736	0.215288	9.12987	6.77922
γ = 0.25	201	179	4.8905	6.6983	0.429606	0.252753	2522	0.197506	22.78712	17.4702
γ = 0.5	182	167	5.2033	5.8503	0.426326	0.237199	2505	0.193781	24.377049	17.7923
γ = 0.75	156	147	5.609	5.1565	0.424706	0.23411	2534	0.198082	27.07	19.8598

**Threshold θ = 0.75**	**Number of clusters**		**Average Size**		**F-score (per output cluster)**		**Running time (sec)**	**Inter-species SS**	**Average number of CE**	**Average number of F-CE**
	**Human**	**Fruit Fly**	**Human**	**Fruit Fly**	**Human**	**Fruit Fly**				
γ = 0	316	264	4.1709	6.2008	0.455754	0.246056	3244	0.20956	9.8042255	7.1234
γ = 0.25	253	230	5.5613	6.1565	0.430593	0.240739	2936	0.194576	28.452756	21.2086
γ = 0.5	233	216	6.5236	5.6343	0.409077	0.228436	2971	0.187672	42.9145	26.1196
γ = 0.75	204	189	6.9608	5.4233	0.404548	0.230347	3569	0.190062	54.52912	29.631

**Threshold θ = 0.5**	**Number of clusters**		**Average Size**		**F-score (per output cluster)**		**Running time (sec)**	**Inter-species SS**	**Average number of CE**	**Average number of F-CE**
	**Human**	**Fruit Fly**	**Human**	**Fruit Fly**	**Human**	**Fruit Fly**				
γ = 0	468	381	4.2671	5.3281	0.435188	0.239389	3694	0.202446	9.670913	6.79617
γ = 0.25	381	338	6.2021	5.5237	0.412203	0.229164	3578	0.18588	47.806789	35.7127
γ = 0.5	333	296	6.955	5.0642	0.394562	0.221976	3551	0.184298	58.4555	38.1736
γ = 0.75	287	260	7.4181	5.0654	0.398062	0.216842	3608	0.180032	78.482639	50.1909
