# Supplementary material for: LePrimAlign: local entropy-based alignment of PPI networks to predict conserved modules
Source: BMC Genomics. 2019 Dec 24;20(Suppl 9):964. doi: 10.1186/s12864-019-6271-3 (PMC6929407; doi:10.1186/s12864-019-6271-3)
Supplement: Supplementary file 3 — Additional file 3 Comprehensive evaluation results of LePrimAlign for yeast and fruit Fly PPI network alignment. The proposed LePrimAlign algorithm has been implemented by changing the parameter values: the threshold θ and the scoring parameter γ. Complex prediction accuracy and alignment quality including inter-species semantic similarities (ISS), the average number of conserved edges (CE) and the average number of functionally consistent conserved edges (F-CE) are shown. [file 12864_2019_6271_MOESM3_ESM.pdf]

Additional File 3. Comprehensive evaluation results of LePrimAlign for yeast and fruit fly PPI network alignment.

Threshold θ = 2	Number of clusters		Average Size		F-score (per output cluster)		Running time (sec)	Inter-species SS	Average number of CE	Average number of F-CE
	Yeast	Fruit Fly	Yeast	Fruit Fly	Yeast	Fruit Fly				
γ = 0	4	4	7	7.75	0.385854	0.366234	47	0.245372	0.25	0.25
γ = 0.25	5	5	4.4	6.6	0.30838	0.335787	46	0.2424	0.2	0.2
γ = 0.5	5	5	5.4	5	0.30838	0.34132	46	0.245576	0.2	0.2
γ = 0.75	5	5	5.6	6.2	0.289643	0.342879	46	0.196144	0	0

Threshold θ = 1.5	Number of clusters		Average Size		F-score (per output cluster)		Running time (sec)	Inter-species SS	Average number of CE	Average number of F-CE
	Yeast	Fruit Fly	Yeast	Fruit Fly	Yeast	Fruit Fly				
γ = 0	6	6	6	6.333	0.479458	0.340109	87	0.210246	0.33333	0.33333
γ = 0.25	6	6	5.6667	6.1667	0.483515	0.323734	91	0.223204	0.33333	0.33333
γ = 0.5	6	6	5.1667	6	0.34813	0.3511	94	0.223308	0.33333	0.33333
γ = 0.75	5	5	5.8	6.8	0.394815	0.358852	89	0.217982	0.2	0.2

Threshold θ = 1	Number of clusters		Average Size		F-score (per output cluster)		Running time (sec)	Inter-species SS	Average number of CE	Average number of F-CE
	Yeast	Fruit Fly	Yeast	Fruit Fly	Yeast	Fruit Fly				
γ = 0	13	13	4.3077	11.7692	0.412637	0.338855	192	0.182273	0.153846	0.15384
γ = 0.25	11	11	4	8	0.397069	0.245907	180	0.244514	0.272727	0.272727
γ = 0.5	12	12	3.9167	7.5	0.397313	0.2375559	183	0.245613	0.272727	0.272727
γ = 0.75	11	11	3.9091	4.6364	0.392054	0.207532	190	0.203265	0.181818	0.181818

Threshold θ = 0.75	Number of clusters		Average Size		F-score (per output cluster)		Running time (sec)	Inter-species SS	Average number of CE	Average number of F-CE
	Yeast	Fruit Fly	Yeast	Fruit Fly	Yeast	Fruit Fly				
γ = 0	23	23	3.4348	9.2609	0.358311	0.316524	243	0.223709	0.434783	0.34782
γ = 0.25	20	20	3.65	5.95	0.403038	0.233048	228	0.223852	0.35	0.25
γ = 0.5	22	22	3.7273	5.8636	0.405167	0.252763	228	0.222056	0.318182	0.22727
γ = 0.75	18	18	4	4.5	0.402427	0.221019	236	0.221624	0.27778	0.16666

Threshold θ = 0.5	Number of clusters		Average Size		F-score (per output cluster)		Running time (sec)	Inter-species SS	Average number of CE	Average number of F-CE
	Yeast	Fruit Fly	Yeast	Fruit Fly	Yeast	Fruit Fly				
γ = 0	37	36	3.3514	9.4722	0.409136	0.317441	329	0.2672	2.216216	2.1081
γ = 0.25	36	35	3.667	7.8	0.426164	0.265532	321	0.25552	3.86111	3.7222
γ = 0.5	33	32	3.6061	5.2188	0.40978	0.248587	328	0.272905	4.35483	4.25806
γ = 0.75	29	28	3.6897	4.5714	0.383355	0.240743	318	0.247944	0.413793	0.31034

Threshold θ = 0.1	Number of clusters		Average Size		F-score (per output cluster)		Running time (sec)	Inter-species SS	Average number of CE	Average number of F-CE
	Yeast	Fruit Fly	Yeast	Fruit Fly	Yeast	Fruit Fly				
γ = 0	37	36	3.3514	4.5878	0.355553	0.254283	878	0.266935	3.349315	3.13698
γ = 0.25	117	112	4.4359	4.6696	0.348337	0.235469	830	0.236243	12.77686	12.5041
γ = 0.5	111	106	4.4865	5.4057	0.340613	0.23369	795	0.232566	11.912281	11.5087
γ = 0.75	74	71	4.2297	5.3521	0.3659	0.25928	834	0.229229	4.855263	4.57894
